# Climate change and specialty coffee potential in Ethiopia

**DOI:** 10.1038/s41598-021-87647-4

**Published:** 2021-04-14

**Authors:** Abel Chemura, Bester Tawona Mudereri, Amsalu Woldie Yalew, Christoph Gornott

**Affiliations:** 1 Potsdam Institute for Climate Impact Research (PIK), Member of the Leibniz Association, Potsdam, Germany; 2 Department of Animal and Wildlife Science, Midlands State University, Gweru, Zimbabwe; 3 International Center of Insect Physiology and Ecology (ICIPE), Nairobi, Kenya; 4 Ca’ Foscari University of Venice & Euro-Mediterranean Center on Climate Change, Venice, Italy; 5 Agroecosystem Analysis and Modelling, Faculty of Organic Agricultural Sciences, University of Kassel, Kassel, Germany

**Keywords:** Biogeography, Plant ecology, Climate-change ecology

## Abstract

Current climate change impact studies on coffee have not considered impact on coffee typicities that depend on local microclimatic, topographic and soil characteristics. Thus, this study aims to provide a quantitative risk assessment of the impact of climate change on suitability of five premium specialty coffees in Ethiopia. We implement an ensemble model of three machine learning algorithms to predict current and future (2030s, 2050s, 2070s, and 2090s) suitability for each specialty coffee under four Shared Socio-economic Pathways (SSPs). Results show that the importance of variables determining coffee suitability in the combined model is different from those for specialty coffees despite the climatic factors remaining more important in determining suitability than topographic and soil variables. Our model predicts that 27% of the country is generally suitable for coffee, and of this area, only up to 30% is suitable for specialty coffees. The impact modelling showed that the combined model projects a net gain in coffee production suitability under climate change in general but losses in five out of the six modelled specialty coffee growing areas. We conclude that depending on drivers of suitability and projected impacts, climate change will significantly affect the Ethiopian speciality coffee sector and area-specific adaptation measures are required to build resilience.

## Introduction

Agricultural commodities face substantial risk from climate change because of their sensitivity to and dependence on weather variables^1^. One such commodity is coffee, a crop and beverage of importance in international trade. The two species of coffee with economic importance are the Robusta coffee (*Coffea canephora* Pierre) and Arabica coffee (*Coffea arabica* Linnaeus). Arabica coffee has relatively higher demand (over 70% of the world coffee market) due to its higher beverage quality^2^. Worldwide, there are five key agroecological zones suitable for producing Arabica coffee, classified on the basis of temperature and rainfall characteristics. These areas are between latitude 20° N and 25° S at altitudes ranging between 700 and 2000 m.a.s.l^3^. Arabica coffee is more sensitive to climatic factors than robusta coffee and thus is expected to be affected more by climate change^4^. This is because Arabica coffee is grown in specific climatic and biophysical envelopes coupled with a narrow genetic diversity^5^. As such, there is evidence that climate change is reducing area suitable for coffee^4,6,7^, limiting yield^8,9^, and increasing the risks of pests and disease^10–12^. These biophysical impacts eventually impinge on the livelihoods of 25 to 30 million smallholder coffee farmers who produce the majority of the world’s coffee^13^.

In addition to the general requirements for Arabica coffee production, and perhaps most importantly, coffee quality profiles are strongly influenced by local climatic (rainfall, temperature, humidity and radiation), topological (elevation, slope angle and aspect), and edaphic (soil depth, acidity/alkalinity and fertility) factors^14,15^. These give the coffee distinctive characteristics specific to production areas. The combination of these factors is unique to each region and thus difficult to replicate, and slight modifications will affect the eventual profile of the coffee, but impact studies on this important aspect are missing. The effect is such that even when the same coffee variety is planted in different areas, the profiles will be different^16–18^. This *terroir* influence in coffee is so strong that it can be detected by chemical traces^18–20^. As such, grading and classification of coffee on the global market are based on the roast appearance and cup quality (flavour, flagrance, acidity and body), bean physiognomies (size, shape and colour), the density of beans and number of defects^21,22^. All of these characteristics are heavily influenced by the geographic characteristics (climate, altitude and soils) of the area of cultivation, the botanical variety and to some extent the preparation (washed or unwashed)^23–25^. In addition, the use of geographical indicators of origin as proxies of product and process quality have grown immensely in the single-origin coffee markets^26,27^. The demand for specialty coffee is increasing across world markets, especially as they are used also in blending with lower quality coffees to create instant coffees. This creates opportunities for coffee growing countries and smallholder communities to receive a premium price of about + 20 to + 50% compared to regular coffee beans^28,29^.

Despite the fact that coffee profiles are dependent on specific climatic and biophysical conditions, there are limited studies on the impacts of climate change on individual coffee types^30,31^. Although it is hypothesised that warming under climate change will reduce coffee quality^23,31^, integrated spatially-explicit quantitative impact studies remain unavailable. For example, it is reported that coffee quality will decrease as mean temperatures rise. This is because the maturation process of the coffee cherry speeds up faster than the development of the bean, leading to lighter and lower quality berries^30^. However, how these translate to potential of specific coffee types is not well established, which in turn makes the associated economic and livelihoods impact assessments difficult.

This study aims to fill this gap. We present evidence of the impact of climate change on the agro-ecological suitability for distinct coffee types, using Ethiopia as a case. Agro-ecological suitability is a measure of the ability of climatic and other biophysical characteristics of an area to sustain a crop production cycle and/or to meet current or expected targets^32,33^. Ethiopia is the largest coffee producer in Africa and the third-largest Arabica coffee producer in the world by volume and value following Brazil and Colombia^34^. Almost all of the coffee is produced by about 5 million smallholder farmers in forest or agroforestry systems, producing an average of 400 000 tonnes with an estimated export value of over US$1 billion^35,36^. In addition, over 10% of the total cropland for commercial agriculture is allocated to coffee production, with coffee exports contributing about a third of all agricultural exports^36,37^. The country also has the largest domestic market for coffee in Africa^38^.

In the elite class of premium single-origin coffees such as Hawaiian Kona Coffee, Indonesian Toraja coffee, or Jamaican Blue Mountain are distinct Ethiopian coffees such as Yirgacheffe, Sidamo, Harar/Mocca, Nekemte, and Limu among others^39,40^. These coffees are recognized in the trade circuits of the world coffee market. These Ethiopian specialty coffees are accorded the best commercial class as “exemplary quality” coffees which have a high intrinsic value with a fine or unique cup and of limited availability compared to the demand of such coffees^21^. Ethiopia is thus a key player in the global specialty coffee industry where the country markets its coffee as distinct based on microclimatic conditions, native heirloom varieties or landraces and other socio-environmental factors^41^. The specific tone/flavour, notes, acidity and body of these are described in Table SI1. Recent studies on climate impacts on coffee show increases in coffee suitability under climate change^4,42^. However, these studies did not consider individual coffee types, with impact studies on wild Arabica coffee showing significant impacts^43,44^.

In this study, we assess the impacts of climate change on five distinct coffee types in Ethiopia. We apply an ensemble modelling approach with three machine learning algorithms driven by six global climate models (GCMs) under four Shared Socio-economic Pathways (SSPs) for four future periods (2030s, 2050s, 2070s and 2090s). We specifically sought to investigate the potential distribution of specialty coffee areas in Ethiopia, identify the important determinants of each specialty coffee, and to quantify the climate change impacts on each specialty coffee. Such climate risk assessment on the specialty coffee sector are imperative to generate scientific evidence on the impacts of climate change on unique economic opportunities for specific geographic regions and most vulnerable communities. In addition to informing policy and trade, this assessment is a first step to identify and undertake, within planetary boundaries, adaptation measures tailored to each coffee type.

## Results

### Model fitting and performance

**Figure 1:**
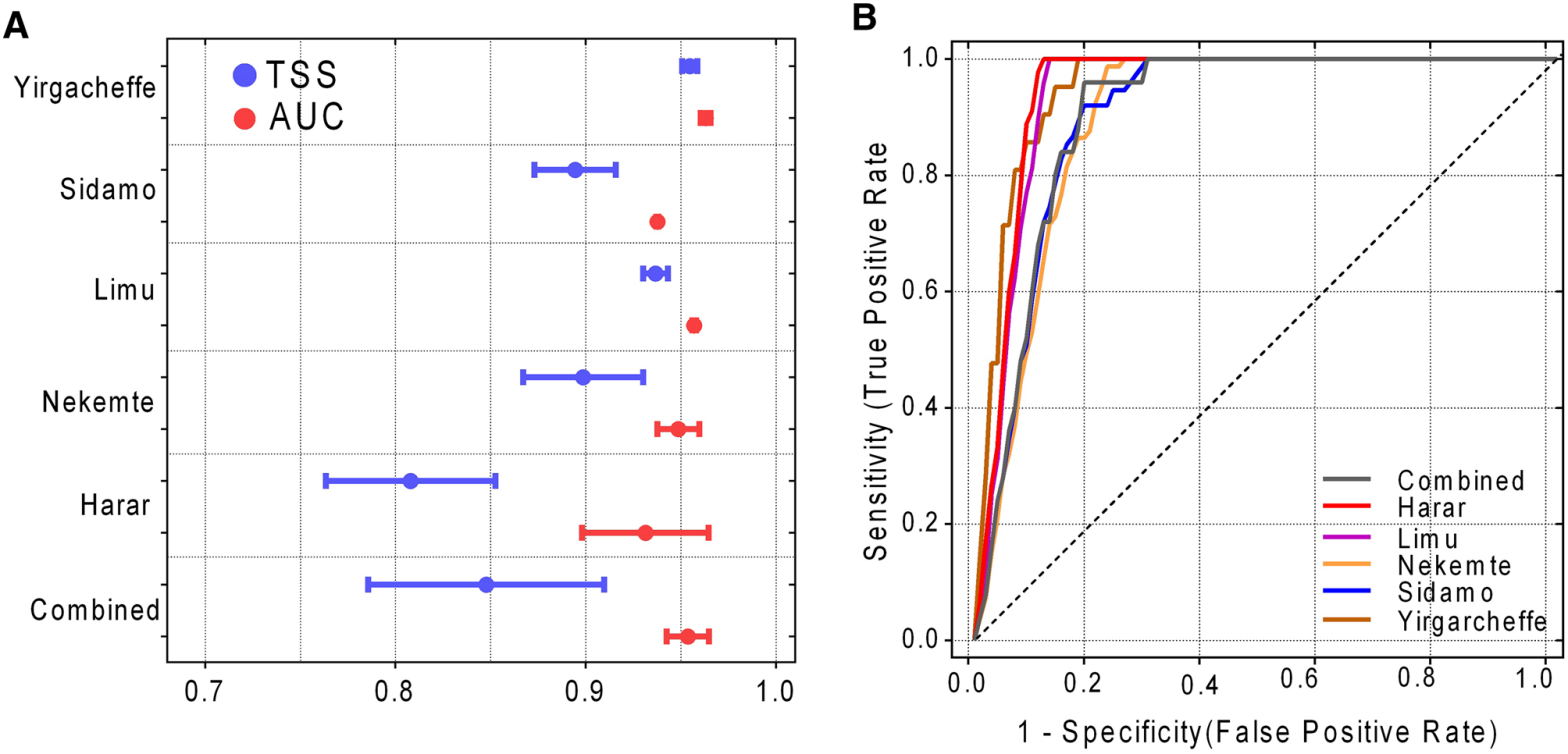
Performance evaluation of the individual models in the ensemble according to (**A**) TSS and AUC (**B**) ROC plots.

The performance of individual models was satisfactory for all the modelling exercises, being exceptional for some specialty coffees (Yirgacheffe and Limu) and high for the rest of the coffees. The lowest AUC and TSS was for Harar model (0.90 and 0.77 respectively), with the combined model for the TSS (0.78) and a higher AUC (0.94). Overall, our model evaluation showed that the modelling of all specialty coffees in Ethiopia was based on model skill and not random chance in identifying coffee areas (Fig. 1). There was a very strong correlation between the area modelled as suitable by the combined model and by the individual coffee models (Figure SI3). The combined model however, slightly overestimated speciality coffee areas in all cases as all specialty coffee types were below the 1:1 line. The high correlation between the combined and individual models, in addition to the high TSS and AUC values provides confidence to apply the model for examining the coffee suitability under current and future climatic conditions.

### Determinants of specialty coffee distribution in Ethiopia

**Figure 2:**
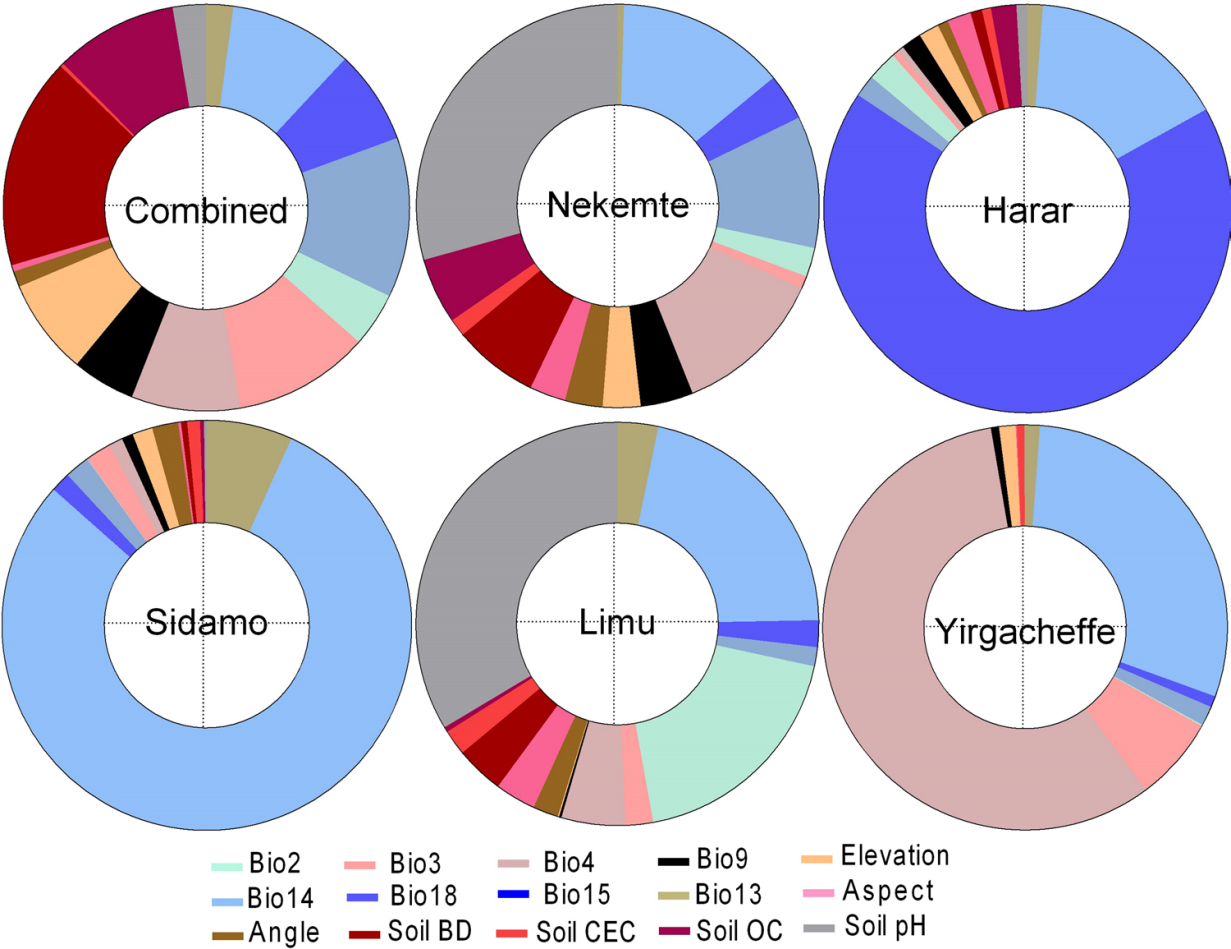
The importance of a variable in explaining coffee suitability for all coffee and five specialty coffees in Ethiopia. Data is obtained from averages of the three individual models. Note the ‘Bio’ variable notations are following the notations used in the WorldClim database, our original database for bioclimatic variables. See SI3 Table for full variable names.

The importance of climatic, soil and topographical factors driving the suitability of coffee production in Ethiopia is shown in Fig. 2. The figure indicates that for the combined model, no specific individual factors dominate suitability whereas there are dominant variables driving suitability for each specialty coffee. Climatic factors are more important in determining the suitability of coffee in Ethiopia in general (61:39) and for the suitability of all specialty coffees except for Nekemte coffee (48:52) (see also Fig. 2), which is influenced by more by soil factors. The most important factor in determining suitability for all coffee in Ethiopia is soil bulk density (BD) (16.8%) followed by precipitation seasonality (Bio15) (12.7%), with coffee preferring areas with lower soil BD and precipitation coefficient of variation (CV). Isothermality (Bio3) (10.8%) also contributed to coffee suitability among climatic variables (Fig. 2). Soil organic carbon (OC) (9.8%) and elevation (7.6%) are also important topographical and soil determinants of coffee suitability while the least important were soil CEC (0.3%) for soil factors, and slope aspect (0.5%) for topographical factors and precipitation of wettest month (Bio13) for climatic factors (2.5%).

Unlike for all coffee suitability, one variable contributed at least more than 25% to specialty coffees suitability in Ethiopia. For example, for Harar coffee, 67% of its suitability of the model fit is influenced by precipitation of warmest quarter (bio18), 79% of Sidamo coffee by precipitation of driest month (Bio14) and 57% of Yirgacheffe coffee by temperature seasonality (Bio4). Conversely, Limu (34%) and Nekemte (29%) coffee distribution are more explainable by soil pH, with temperature seasonality (Bio4) the second most important for both coffees (21 and 14% respectively, Fig. 2). The variation of the factors influencing the suitability of all coffee and specialty coffee indicate that there are localized factors that explain individual coffee suitability, after general factors that determine the suitability of coffee in Ethiopia.

### The geographic distribution of specialty coffee areas in Ethiopia

**Figure 3:**
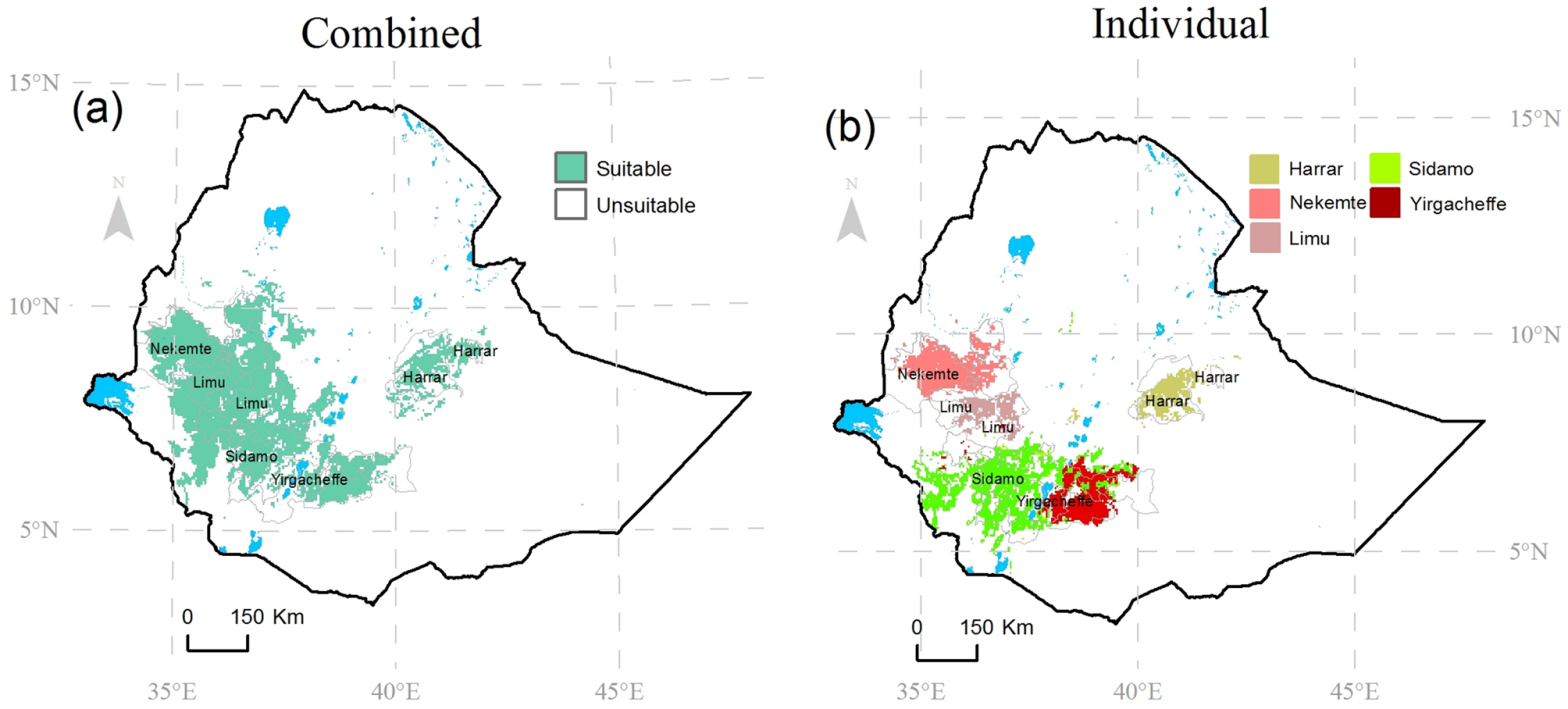
Suitability for coffee production under current climatic conditions in Ethiopia modelled with the (**a**) combined model and (**b**) individual specialty model. The model results were exported into ArcGIS Sofware Version 10.2 (http://desktop.arcgis.com/en/arcmap) to generate the map in this figure.

The combined model shows that 27% (299,193 km^2^) of Ethiopia is suitable for coffee under current climatic conditions, with the highest area (63%, which is 188,652 km^2^) outside the five specialty coffee zones (Fig. 3a, Table 1). Of the 27% that is suitable for coffee in Ethiopia, only about 30% (91,122 km^2^) is suitable for specialty coffee production. The individual specialty coffee models show that the Nekemte coffee has the largest suitable area (32,648 km^2^, 11.8%) in the western parts of the country, followed by Sidamo coffee with 9.6% of the suitable area for coffee (26,478 km^2^) in the southern parts of the Ethiopian highlands. The smallest range is for Yirgacheffe coffee which is only suitable in 1.2% of the suitable area for coffee in the combined model (3,736 km^2^, Table 1). The most significant overlaps were for Sidamo and Yirgacheffe coffee where 65% (2411 km^2^) of the Yirgacheffe was modelled as suitable for Sidamo coffee while 16% (5,170 km^2^) of the Sidamo coffee area was modelled as suitable for also producing Yirgacheffe coffee (Fig. 3, Table 1).

**Table 1:** Area and percentage suitable for growing coffee under current climatic conditions.

Coffee typicity	Combined model		Individual models	
	Area (km^2^)	Percentage	Area (km^2^)	Percentage
Harar	18,029	6.0	13,120	14.4
Nekemte	38,165	12.8	32,648	35.8
Limu	19,050	6.4	15,944	17.5
Sidamo	31,562	10.5	26,479	29.1
Yirgacheffe	3736	1.2	2932	3.2
Other	188,652	63.1	n/a	n/a
Total	299,193	100	91,122	100

### The impacts of climate change on specialty coffee in Ethiopia

**Figure 4:**
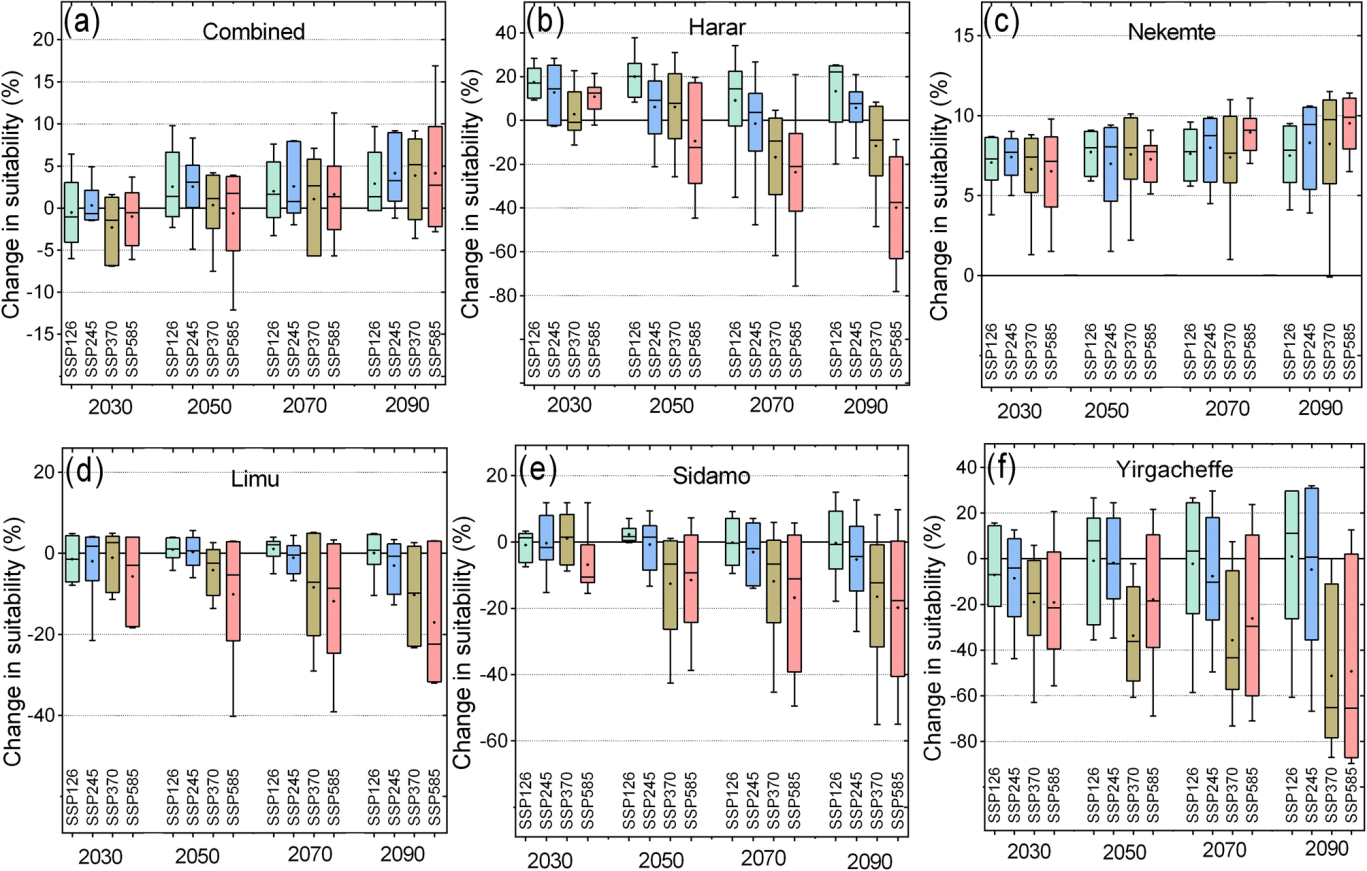
Simulated impacts of climate change on specialty coffee suitability in Ethiopia by 2030s, 2050s, 2070s and 2090s. The bar plots show the range of projected changes using the ensemble model and the variability from the six GCMs.

The impacts of climate change on each specialty coffee and the combined effects are shown in Fig. 4. The results show that the area that is suitable for coffee in the country will increase gradually until 2090s from the combined model (Fig. 4a). In the near future (2030s), the area suitable for coffee will remain stable under all scenarios (less than 1% average change in suitable area). For the 2050s, under SSP126 and SSP245 scenarios, the area suitable is projected to increase on average by 2.9% and 2.3% respectively but remain stable under SSP370 scenario (+ 0.4%) and decrease in the SSP585 scenario (− 1.3%). The area suitability is projected to increase by the 2070s and 2090s under all SSPs. Except for SSP370 where coffee suitability is projected to remain stable, it is projected that coffee suitability will increase on average in the 2070s by 2.3, 3.0 and 1.5% under SSP126, SSP245 and SSP585 respectively. The highest average increase of 4.5% is projected under SSP245 in 2090s (Fig. 4a, Table SI3).

**Figure 5:**
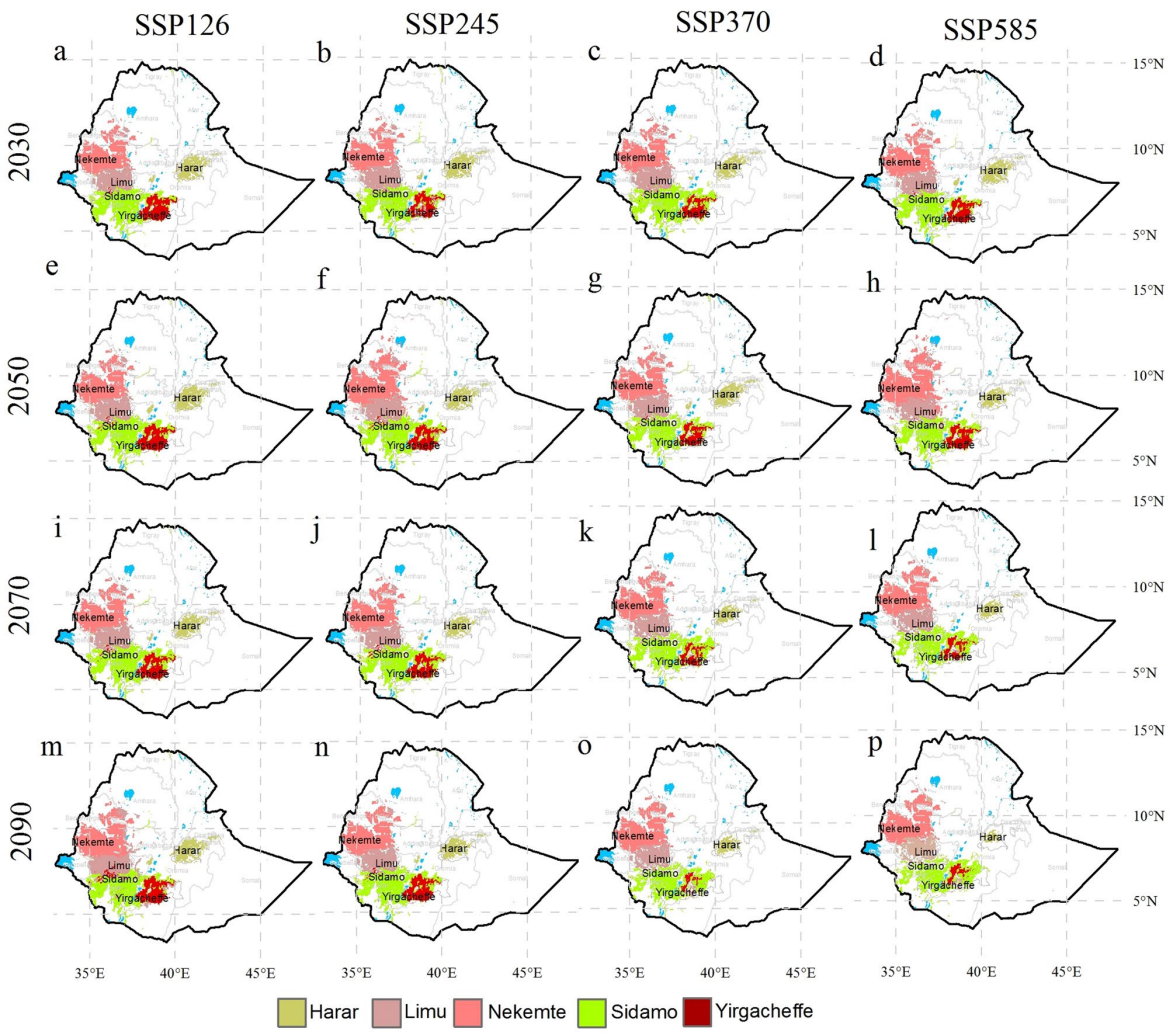
Maps showing the projected changes in the area suitable for growing specialty coffee in Ethiopia in the 2030s, 2050s, 2070s and 2090s. The results are obtained from four scenarios obtained from the ensemble model. The model results were exported into ArcGIS Software Version 10.2 (http://desktop.arcgis.com/en/arcmap) to generate the map in this figure.

Depending on the scenario and the time-period, the area suitable for all specialty coffee types is projected to decline under climate change except for Nekemte, which is projected on average to increase its suitable area (Figs. 4b–f, 5). Largest decrease in the suitable area are projected for the Harar and the Yirgacheffe coffees which are projected to lose, on average, more than 40% of their suitable areas by 2090s under the worst case scenario (SSP585) (Fig. 5, Table SI3). The projected decreases in the area suitable for specialty coffee in Ethiopia contrast the results of the combined general model which shows an increase. The country-level change in the area suitable is, therefore, more influenced by the Nekemte coffee which has the highest area suitable for coffee under current climatic conditions. This implies that the national-level results mask the impacts of climate change on the suitability of specialty coffee in Ethiopia. In other words, country-level studies may reveal little about region-specific socio-economic consequences of ensuing impacts of climate impacts on specialty coffees.

## Discussion

The impacts of climate change on the quality and quantity of coffee production has gained recent attention. Nevertheless, previous studies have hardly addressed the impacts of climate change on specialty coffee, an important aspect of the global coffee sector. In this study, we developed an ensemble model to project the suitability of specialty coffee in Ethiopia under climate change, and thus to quantitatively and spatially gauge the impacts of climate change on the coffee types. For this purpose, five specialty coffee types—Harar, Limu, Nekemte, Sidamo and Yirgacheffe—were modelled under current and future climatic conditions.

Our study provides further specific information about climate change impacts on the coffee sector by looking at specific coffee systems. This is an advancement from previous studies which were limited to the application of suitability models to understand the geographic range and climate change impacts on the overall coffee sector^6,44–47^. Our projected area of coffee production match the observed and previously reported ranges for coffee production in Ethiopia including the original habitats and the primary and secondary production areas on either side of the rift valley^42,48,49^. Our finding that only about 30% of the coffee producing area is suitable for specialty coffees concurs with current estimates that around 20% of coffee production in Ethiopia is specialty coffee^50^. Assuming similar yield levels between general and specialty coffee, in line with the generalist nature of suitability models, we believe our suitable area simulations are therefore very reliable.

There are no verifiable records about coffee production amounts or ranking according to each specialty type. Yet, with the decent match of our combined and individual model in projecting these areas, we conclude that we have provided a robust estimate of the potential areas for the aforementioned coffee types. We, however, notice an increase of projected coffee suitability in the south-western part of the country which is beyond current established production areas. This is expected as the suitability maps capture the potential production areas, some of which have not yet been used as coffee production areas.

We find that the factors that influence the suitability of coffee, in general, vary with those that give geographical typicality. We find that there is an almost equal contribution of temperature and precipitation factors in determining the suitability of coffee in Ethiopia, confirming findings from other studies^42^. Conversely, in a global study, temperature factors were identified mainly as determinants of Arabica coffee suitability^4^ while in a national study elsewhere identified mostly precipitation-based factors in determining suitability^6^. Such variations are explained by differences in scale and geography respectively, indicating that the potential for coffee can be influenced by local and regional factors. This explains our finding that specialty coffee types are influenced by different factors giving them different chemical properties and profiles. For example, Tolessa, et al.^51^ finds that coffee specialty cup quality attributes (overall cup preference, acidity, body, flavour and aftertaste) were influenced more by altitude. It is important to note that the altitude is not a direct production parameter for coffee but strongly influences climatic and soil characteristics that in turn influence the coffee quality. The reasons for higher quality at higher altitude is the slow coffee bean development associated with cooler high-altitude temperatures, which allow for slow bean filling and can result in more flavour profiles and body^6^. In addition, coffee cultivars are different for each specialty coffee zone^41,49^ and yet coffee varieties respond differentially to climate change impacts^52^.

Although we include, for the first time in modelling suitability of coffee, soil and topographic factors we find that climatic factors are more important in determining suitability except for the Nekemte coffee in which soil factors are the most important. In addition, we explore the factors explaining the geographical provenance of Ethiopian coffee, confirming that the current branding and individual marking of these specialty coffees is justifiable based on the different climatic, soil and topographic factors that influence them. These distinct influences manifest in the cupping profiles and elements in the coffee from other assessment methods^18–20^. This demonstrates the importance of the different environmental determinants of coffee profiles as similarly reported for wine quality^53^. Chemometric methods have, for example, shown that environmental conditions during the development of coffee beans have a strong influence on the distribution of coffee bean fatty acid composition, an important indicator of quality^54^. This further strengthen the need for protecting and promoting coffees that are linked to origin and not just to postharvest processimg. This also underlines the importance of assessments specific to individual coffee types calling for more localized climate impact studies compared to general ‘all-in-one-basket’ approaches.

The results show that we can expect an overall increase in coffee growing potential in Ethiopia but a decrease in most specialty coffees (except Nekemte) by climate change from the near-future to the 2090s. Similar positive changes in general coffee suitability in Ethiopia were reported^4,42^. On the other hand, studies that focused on wild coffee Arabica reported decreases of up to 50% in areas suitable in Ethiopia^42,44^, implying the possible differences in suitability in wild and cultivated distribution of coffee in Ethiopia. The implications of changes in wild Arabica coffee suitability attribute more to losses in genetic diversity which may affect future breeding programs while losses in production area suitability for cultivated coffee are directly related to socio-economic factors. Specifically, losses in the production potential of specialty coffee indicate losses in lucrative specialty markets as coffee move into generic (bitter) coffee categories in terms of quality profiles (e.g. similar production potential can be achieved but with a changed taste profile).

The overall impact of lost specialty status is lost market premiums, which disincentives some farmers from continuing with specialty coffee production as they move to alternative crops. In many cases once the specialty status of the coffee has been lost it is difficult for the farmers to continue viably producing the coffee because they will not be able to compete with industrial coffee production systems elsewhere that are more efficient but produce generic coffees. This will have impacts on both the local and national economy. Our study concurs with some estimates that up to half of the current specialty coffee growing areas could be significantly altered by climate change with effects on production and local society^35,42,55^. Broadly, this study underscores the importance of understanding the dimensions of impacts of climate change in impact assessments. We conclude that rainfall increases may favour coffee production in general but changes in specific climatic characteristics has more significant effect on specialty coffee types and this has significant and downstream impacts on the local and global coffee sector. Therefore, impact studies should also consider such dimensions.

Some studies show that coffee can respond positively to carbon CO_2_ fertilization^5,56^. Nevertheless, it is difficult to account for interactions between elevated CO_2_ and environmental conditions in empirical models such as those used in this study. CO_2_ fertilization may expand (or offset) the increases (or the decrease) in suitability by an unknown extent. Besides, climate change impact was calculated as the change in area suitable relative to the current (baseline) and not as absolute change. In addition, smaller or emerging specialty areas that were not represented in the modelling points could have been under-estimated in our study. Crop suitability modelling also allocates similar weight to all the points and yet these points may vary in their suitability from highly suitable to marginally suitable, which is not reflected in this study.

We believe that results from this study provide a scientific evidence which underpins both national and subnational adaptation planning for a climate-resilient speciality coffee sector. The fact that the information is spatially explicitly also means that various adaptation measures can be suggested for different areas thereby reducing the risks of maladaptation. For example for coffee types where temperatures are most important, enhanced agroforestry systems can be suggested to regulate canopy temperature while irrigation is important for those where rainfall factors are most important. Future research should integrate the results of such studies with economic models to get a better picture on both the biophysical and economic consequences (e.g., on employment, sectoral and regional GDP, export earnings and trade balances) of climate change-induced area suitability changes on speciality coffees in the country.

## Conclusions

In this study, we apply an ensemble of three models the current and future suitability of specialty single-origin coffees in Ethiopia to understand and identify areas and opportunities for adaptation. Three important conclusions can be drawn from the study results. First, there are differences in factors determining all coffee suitability and specialty coffee suitability with factors influencing each type being different among specialty zones. Second, the projected gain in coffee suitability is highly influenced by changes in the largest specialty zone as five out of the six modelled zones are projected to lose their suitability for their respective specialty coffee type. Third, the magnitude of the impacts of climate change depends on the radiative forcing (GHG emission) scenario and socio-economic pathway and the time period with worst results expected under the highest emission scenario (RCP8.5) around the end of the century (i.e., 2090s). We conclude that the specialty coffee sector faces production risks from climate change but there are opportunities for adaptation strategies to build resilience for the sector.

## Methods

### Coffee farm locations

We first obtained and digitized maps of the specialty coffee production areas in Ethiopia^48^. We then identified 8 specialty coffee types, namely, Bale, Guji, Harar, Keffa, Nekemte, Limu, Sidamo and Yirgacheffe. Then, we compiled GPS coordinates of coffee farms in Ethiopia from the following four sources:

- CABI Crop Protection Compendium, (CABI-CPC, https://www.cabi.org/cpc/): The compendium is an encyclopaedic resource with a collection of science-based information on all aspects of crops that have been sourced from experts, independent scientific organizations, and specialist organizations in the form of images, maps, and geographic coordinates^57^. We, therefore, searched for reports on “*Coffea arabica*” for Ethiopia and retrieved all coordinates.
- Global Biodiversity Information Facility (GBIF, www.gbif.org): The GBIF was established in 2001 to publish primary biodiversity data using community-driven and agreed standards and tools. It facilitates open access to biodiversity data worldwide for scientific research, conservation and sustainable development with over one billion occurrence records of species.
- Integrated Digital Collection (iDigBio, www.idigBio.org): iDigBio is an online resource for specimen digitization and digital data mobilization for researchers to visualize, analyse, and model species with possibilities for big data strategies. Millions of specimen-based occurrence data are available on this portal.
- Scientific publications: To supplement the presence points obtained from the databases, coffee points were also digitized from two scientific publications that have coffee presence points for Ethiopia. These were Ridley^38^ and Moat, et al.^42^.

We cleaned the data by removing records (1) without or with incorrect geographic coordinates, (2) older than the year 2000 since they may have positional errors (3) with a reported uncertainty higher than 50 km, and (4) within 5 km of another point to avoid extracting same pixels with same points.

After these steps of data cleaning, a total of 267 valid geographic points were obtained and used for the modelling. Using the specialty coffee zone shapefile, the points were clipped to each specific zones for suitability modelling of specialty coffee and all points used together without distinction for the *combined model* (Figure SI1). We further did an area-weighted elimination of presence points so that the modelling is not biased by some specialty areas having more points than other areas. We were finally left with only five coffee specialty zones which are Harar, Nekemte, Limu, Sidamo and Yirgacheffe (see also Table SI1). The use of these presence points is based on the assumption that coffee farmers grow coffee in the most suitable agro-ecological areas with varieties/landraces and production systems significantly different for each specialty coffee type. There are between 6000 and 10,000 regional heirloom coffee varieties in Ethiopia in addition to the over 40 hybrids that have been developed for each region mostly for higher yields and pest and disease resistance. The modelling captures distribution of these genetic material as points from each coffee speciality zone represent specific varieties that vary between regions. Therefore, the modelled suitability for the coffee types is the ability of a region’s climatic and other conditions to sustain production of current varieties and achieving current production levels.

Fitting the suitability models uses the presence points and background absence data. The coffee presence points provided the locations of coffee farms and there was a need to sample for background absence data. We developed a background sampling protocol that (a) gives a balance between the numbers of absences relative to the number of presences in the modelling dataset as this leads to overfitting and inflation of model performance metrics, (b) limits the loss of important information, and (c) preserves the observed prevalence. To achieve this, we settled for a presence to absence ratio of 1:10 in the dataset for the combined model and the individual specialty coffee models which is also recommended in literature^58,59^. The background sampling was applied outside a buffer of 10 km of presence points to limit them to the outside known coffee areas.

### Agro-ecological variables for suitability modelling

Three types of agro-ecological variables were used in modelling the current and projected suitability of distinct specialty coffee types in Ethiopia. These were climatic variables, topographical variables and soil variables. The variables were selected on their known agronomic significance to coffee production.

### Climatic variables

The 19 climatic variables available from the WorldClim v2.1 database (www.worldclim.org) at ~ 5 km × 5 km spatial resolution were downloaded and clipped for Ethiopia. These variables are derived from monthly values of precipitation and temperature throughout the year into agronomically and ecologically useful variables for primary production-based species modelling. The WorldClim database curates these data interpolated from more than 115,000 climate stations from the global climatic database such as FAOCLIM, the Global Historical Climate Network (GHCN) and other sources^60^. The climatic variables, therefore, represent annual tendencies such as average annual temperature and precipitation values and their variations, seasonal characteristics such as temperature and precipitation ranges in the wettest, driest, hottest, and coldest quarters and extremes such as temperature in the coldest month or precipitation in the wettest month. Detailed information of each variable are given in the Table SI2.

### Topographical variables

Three topographic variables (elevation, slope angle and aspect) were included in the modelling as these also influence coffee suitability and local characteristics of specialty coffee types. The GTOPO30 global digital elevation model (DEM) was downloaded from United States Geological Survey (USGS) for deriving the topographic variables. The GTOPO30 was developed by the USGS’s EROS Data Centre (EDC) and provides elevations at 30 arc-seconds (~ 1 km). In addition to using the elevation (m.a.s.l) as a variable, slope aspect (1–12 directional values) and slope angle (degrees) were derived from the DEM as additional variables. We hypothesised that, since the amount of radiation received varies depending on the direction the slope is facing, this may influence the development of distinct characteristics of the coffee. We also hypothesised that slope angle has an influence on water accumulation and soil depth which in turn may influence not just the suitability of coffee but the development of its specific profile. Slope and angle variables were derived using the R programming language^61^ after the variables were aggregated to the resolution of the climatic variables.

### Soil variables

In addition to the climatic and topographic variables, four soil variables were also included. These were soil pH (has a measure of soil acidity or alkalinity levels), soil cation exchange capacity (CEC) soil apparent bulk density (BD) and soil organic carbon (OC). These were obtained from the International Soil Reference and Information Centre (ISRIC) Africa soils database that is a 1-km soil map of properties and soil classes obtained from advanced regression analysis of thousands of published and compiled soil profiles from many sources^62^. Soil variables were included because soil type and related factors are important determinants of coffee production^63,64^. The soil variables were also aggregated to the resolution of the climatic variables.

### Collinearity test for variable selection

A stack with a total of 26 variables was therefore created from the climatic, topographic and soil variables. To remove correlated variables (Figure SI2) that do not provide additional unique information to the modelling, the variance inflation factor (VIF) method was used for collinearity test and variable selection and for improving model stability, robustness and computing efficiency. VIF measures the inflation of the variances for the parameters above what is expected if there is less multicollinearity among the independent variables^65^. The VIF is directly calculated from the linear model with the focal numeric variable as response using Eq.(1):

1 $$VIF = \frac{1}{{1 - { }R_{i}^{2} }}$$

where R^2^ is the regression coefficient of determination of the linear model. A VIF value greater than 10 often suggests a collinearity problem within a model, and therefore 10 is used as the elimination threshold^66^.

### Modelling approach

We built and applied an ensemble modelling approach to deal with uncertainties involved in individual model performance and prediction. After the exploratory model runs, three machine learning algorithms, Random Forest (RF), Boosted Regression Trees (BRT) and Support Vector Machine (SVM) outperformed 15 other algorithms in terms of accuracy and were used for building the ensemble model.

### Random forest

Random forest is an ensemble classifier that consists of many decision trees developed by Breiman^67^. The algorithm operate by constructing a multitude of decision trees at training time and outputting the class that is the mode of the classes or mean prediction of the individual trees while internally correcting for decision trees' habit of overfitting to their training set. Thus, RF is not sensitive to the problem of overfitting as it can handle large datasets and runs efficiently without variable deletion^67^. Random forest is therefore one of the most accurate learning algorithms with high performance in predicting species distributions.

### Boosted regression trees

Boosted regression trees (BRT) is a machine learning that combines boosting method with classification and regression trees (CART). BRT combine the strengths of regression trees (models that relate a response to their predictors by recursive binary splits) and boosting (an adaptive method for combining many simple models to give improved predictive performance)^68^. The final BRT model is as an additive regression model in which individual terms are simple trees, fitted in a forward, stage wise fashion with much better prediction performance than its weaker predecessors^69,70^.

### Support vector machine

The SVM is a universal machine learning method for cogent prediction based on structural risk minimization and statistical learning theory^71^. The SVM algorithm maps the original data into a high dimensional feature space where a hyperplane is constructed from training data and uses a kernel function to transform the data into a SVM. Samples located in the boundaries (support vectors) are identified and used to compute an optimal decision boundary^72^. The optimal linear hyperplane is used in order to separate the original input space^71^.

### Building an ensemble model

An ensemble modelling approach was used. This approach combines predictions from each model and often results in better model predictions than relying on individual models^73^. We used a weighted averaging approach based on Area Under the Curve (AUC) statistics to assign how much each of the three individual models contributes to the final model when its AUC is above 0.75 (Eq. 2):

2 $$E = \frac{{\mathop \sum \nolimits_{i = 1}^{n} (AUC_{i} *M_{i} )}}{{\mathop \sum \nolimits_{i = 1}^{n} AUC_{i} }}$$

where, E is ensemble, AUC_i_ is the AUC value of the *i*th single model (M_i_).

Finally, we used the *predict* function of R to project the models both to current environmental conditions across the study area and to future environmental scenarios for each model for Ethiopia. The suitability maps were converted into binary suitable and unsuitable based on the specificity-sensitivity sum maximization approach.

### Variable importance

Variable importance was calculated to determine to what extent each predictor variable contributes to the predictions made by the model. The variable importance of each predictor was extracted from each SDM. This method is a randomization procedure that measures the correlation between the predicted values of a model with the original predictors, and predictions of the same model with a randomly permutated dataset under evaluation. If the contribution of a variable to the model is high, then it is expected that the permutation would affect the prediction, and consequently, the correlation is low and vice-versa. Using this approach, ‘1 –correlation’ is considered as a measure of variable importance and was used to assess the importance of each predictor to the combined coffee model and individual specialty coffee models. The individual variable importance values are dependent on the individual algorithm used, mean values for the three models were calculated to provide relative information on predictor importance within the model.

### Model evaluation

To assess model performance, the modelling data were divided into a training data set (70%) and testing data set (30%). The training data set was used for model calibration, and the testing data set was used for out-of-sample model performance. One hundred replicates were run for each model with resampling methods for subsampling. The area under the receiver operating characteristic curve (AUC), which measures overall discrimination capacity and the *true skill statistic* (TSS), which balances the capacity to correctly predict presences and pseudo-absences, were used to measure the accuracy^74^. A perfect model would have an AUC and TSS of 1. The AUC varies from 0 to 1, while the TSS varies from − 1 to 1, with values above 0.75 showing good model skill for both^75^. All model calibration, evaluation and projection were done with the *sdm-R* package version 1.0–8974^76^. All modelling as much as possible confirmed to recommended standards in species modelling^77^. The model results were exported for producing map figures in ArcGIS 10.6 (Environment Systems Research Institute, Redlands, CA)^78^.

### Assessing the impacts of climate change

One of the advantages of suitability models is that models fitted in current conditions can be transferred to novel periods under emissions scenarios projections to understand and quantify potential variations in species ranges due to climate change. To project future combined and individual coffee suitability, the topographic and soil predictors were considered unchanging, while for independent climatic predictors we used those extracted by six bias-adjusted Coupled Model Inter-comparison Project (CMIP6) available from the WorldClim v2,1 database at a spatial resolution of 2.5 min^79^.

Six GCMs were used for projected climatic conditions. These were Beijing Climate Centre China Meteorological Administration (China, BCC-CSM2-MR), Centre National de Recherché Meteorologiques (France, CNRM-ESM2-1), Canadian Centre for Climate Modelling and Analysis (Canada, CanESM5), Institut Pierre Simon Laplace (France, IPSL-CM6A-LR), and Center for Climate System Research (Japan, MIROC6) and Meteorological Research Institute (Japan MRI-ESM2-0). These GCMS were selected because they have complete bias-corrected data for all the periods that were under consideration (2030s, 2050s, 2070s and 2090s).The bias correction was performed through delta downscaling method following Navaro^80^. In this approach, a change factor or ‘delta’ is derived from the GCM, and then added onto the observations to provide a bias-corrected and high-resolution representation of the mean climates. GCM-based model uncertainty was calculated by determining the number of models agreeing in the prediction of the direction of the climate change trend.

The impact of climate change on specialty coffee in Ethiopia was assessed from projected climate and socio-economic conditions defined based on the combination of the Representative Concentration Pathways (RCPs) and the Shared Socio-economic Pathways (SSPs). The RCPs provide future climate simulation through considering the impact of future greenhouse gas emission trajectories on the climate system^81^. There are four RCPs -RCP 2.6, RCP 4.5, RCP 6.0 and RCP 8.5—on the basis of the forcing until the end of twenty-first century. The SSPs represent a distinct set of narratives about the future of the world through a wide range of plausible trajectories of population growth, economic growth, technological development, trade development and implementation of environmental policies^82,83^. The five SSPs are commonly referred as the most sustainable development (SSP1), middle-of-the-road development (SSP2), regional rivalry (SSP3), inequality (SSP4) and full fossil-fuelled development (SSP5) pathways.

The combination of RCPs and SSPs, therefore, represent a wide range of plausible future scenarios that are more probable by the integration of radiative forcing and socioeconomic development influences^84^, providing a more comprehensive scenario matrix. This is because each SSP is broadly aligned with one or two RCPs, allowing easy integration of SSPs and RCPs. The four combinations chosen were SSP1-RCP2.6 (SSP126), SSP2-RCP4.5 (SSP245), SSP3-RCP7.0 (SSP370) and SSP5-RCP8.5 (SSP585) and these linear combinations were chosen to enable comparison in similar studies that apply the same combinations. We ran the models for four future periods which are 2030s (2021–2040), 2050s (2041–2060), 2070s (2061–2080), and 2090s (2081–2100).

## Supplementary Information

Supplementary information.

## Data Availability

The data that support the findings of this study can be found in the related cited articles and/or from the corresponding author upon reasonable request.

## Code availability

The compiled code and files used in the model setup, validation and projections are freely available for download at GitHub: https://github.com/achemura/Specialty-coffee-Ethiopia.
