# Supplementary material for: Climate change and specialty coffee potential in Ethiopia
Source: Sci Rep. 2021 Apr 14;11:8097. doi: 10.1038/s41598-021-87647-4 (PMC8046822; doi:10.1038/s41598-021-87647-4)
Supplement: Supplementary file 1 — Supplementary information. [file 41598_2021_87647_MOESM1_ESM.docx]

**Climate change and specialty coffee potential in Ethiopia**

Abel Chemura^1*^, Bester Tawona Mudereri^2^, Amsalu Woldie Yalew^1^ & Christoph Gornott^1^

^1^Potsdam Institute for Climate Impact Research (PIK), Member of the Leibniz Association, Potsdam, Germany

^2^Department of Animal and Wildlife Science, Midlands State University, Gweru, Zimbabwe

^3^International Center of Insect Physiology and Ecology (ICIPE), Nairobi, Kenya

^4^Agroecosystem Analysis and Modelling, Faculty of Organic Agricultural Sciences, University of Kassel, Kassel, Germany.

[*chemura@pik-potsdam.de](mailto:*chemura@pik-potsdam.de)

Supplementary Information

**Climate change and specialty coffee potential in Ethiopia**

**Supplementary Information**

**Figure SI1: Coffee points used in the modelling process.** The map was produced in ArcGIS Sofware Version 10.2 (http://desktop.arcgis.com/en/arcmap).


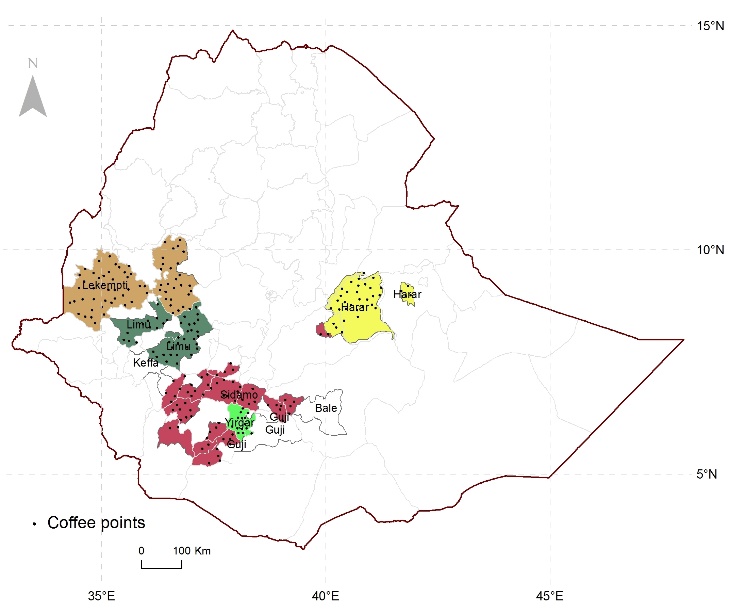


**Figure SI2:** Pearson correlation coefficient of the 24 bioclimatic, topographic and soil variables


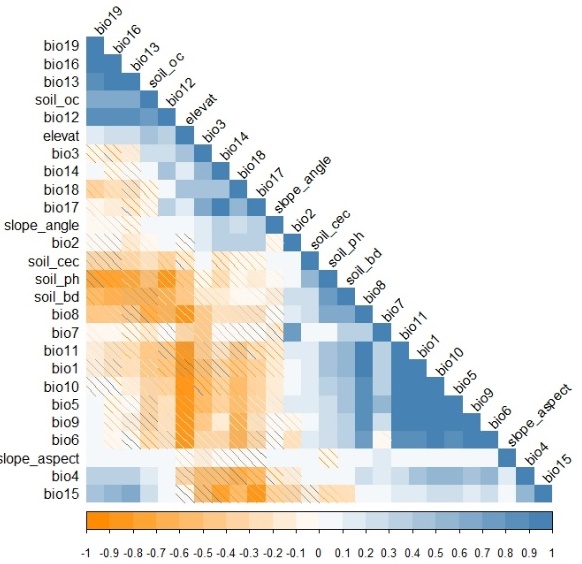


**Figure SI3: Correlation between combined model suitability and individual model suitability area for coffee in Ethiopia under current climatic conditions.**


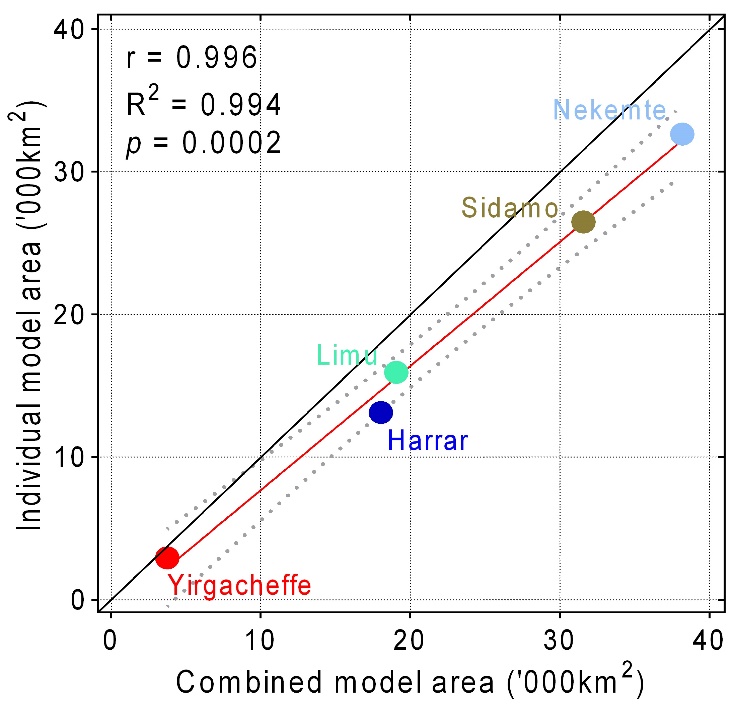


**Table SI1: Characterization of the distinct coffee types in Ethiopia**

| **Type** | **Tone/Flavour** | **Description** | **Acidity** | **Body** |
| --- | --- | --- | --- | --- |
| Harar | Distinctive winey or fruity tones | - Floral and fruity mocca notes often complex shortberry, longberry or peaberry. - Slightly fermented aftertaste | Bright (sometimes brilliant) | Medium to heavy body |
| Limu | Winey and spicy flavours | - Sweet and vibrant winy with floral overtones. - A hint of nut-toned aroma. - Pleasant after taste | Fair light medium acidity | Well-balanced body |
| Nekemte | Slight yet distinct fruity flavour. | - Fruity finish | Pleasant acidity | Healthy body reminiscent |
| Sidamo | Sweet and complex flavour. | - Spicy floral aroma - A finish that is bright and soft - Bright and vibrant aftertaste | Low acidity, | Rich, full body |
| Yirgacheffe | Sweet to spicy fragrant. | - Fragrant floral notes in the aroma, - May exhibit undertones of berry or wine - Often with a hint of toasted coconut or shimmering notes of citrus. | Bright acidity | Light to medium delicate body |

Source: Cupping tests and profiles^1,2^

**Table SI2:** List and description of o bioclimatic variables that were used in the modelling.

| **Code** | **Name** | **Units** |
| --- | --- | --- |
| BIO1 | Annual Mean Temperature | °C |
| BIO2 | Mean Diurnal Range (Mean of monthly (max temp - min temp)) | °C |
| BIO3 | Isothermality (BIO2/BIO7) (×100) | None |
| BIO4 | Temperature Seasonality (standard deviation ×100) | °C |
| BIO5 | Max Temperature of Warmest Month | °C |
| BIO6 | Min Temperature of Coldest Month | °C |
| BIO7 | Temperature Annual Range (BIO5-BIO6) | °C |
| BIO8 | Mean Temperature of Wettest Quarter | °C |
| BIO9 | Mean Temperature of Driest Quarter | °C |
| BIO10 | Mean Temperature of Warmest Quarter | °C |
| BIO11 | Mean Temperature of Coldest Quarter | °C |
| BIO12 | Annual Precipitation | mm |
| BIO13 | Precipitation of Wettest Month | mm |
| BIO14 | Precipitation of Driest Month | mm |
| BIO15 | Precipitation Seasonality (Coefficient of Variation) | Fraction |
| BIO16 | Precipitation of Wettest Quarter | mm |
| BIO17 | Precipitation of Driest Quarter | mm |
| BIO18 | Precipitation of Warmest Quarter | mm |
| BIO19 | Precipitation of Coldest Quarter | mm |

Notes: Bioclimatic variables are climate indices, which highlight climate conditions best related to species growth, physiology, survival and productivity potential. They are derived from the monthly temperature and rainfall values in order to generate more agronomically meaningful variables. The bioclimatic variables represent annual trends (e.g., mean annual temperature, annual precipitation) seasonality (e.g., annual range in temperature and precipitation) and extreme or limiting environmental factors (e.g., temperature of the coldest and warmest month, and precipitation of the wet and dry quarters). A quarter is a period of three months (1/4 of the year). The future data is downscaled and calibrated (bias correction) future climate projections from CMIP6 data.

**Table SI3:** Projected mean percentage changes (of GCMs) in specialty coffee suitability in Ethiopia under different future climate change scenarios compared to the current suitability.

| Period | Scenario (SSP-RCP) | Combined | | Harar | | Nekemte | | Limu | | Sidamo | | Yirgacheffe | |
| --- | --- | --- | --- | --- | --- | --- | --- | --- | --- | --- | --- | --- | --- |
|  |  | Area (km^2^) | % | Area (km^2^) | % | Area (km^2^) | % | Area (km^2^) | % | Area (km^2^) | % | Area (km^2^) | % |
| 2030s | SSP126 | -3115 | -1.0 | 2284 | 17.4 | 2302 | 7.1 | -224 | -1.4 | -232 | -0.9 | -210 | -7.2 |
|  | SSP245 | 1673 | 0.6 | 1673 | 12.7 | 2422 | 7.4 | -311 | -2.0 | -83 | -0.3 | -250 | -8.5 |
|  | SSP370 | -6178 | -2.1 | 377 | 2.9 | 2169 | 6.6 | -170 | -1.1 | 308 | 1.2 | -558 | -19.0 |
|  | SSP585 | -4036 | -1.3 | 1416 | 10.8 | 2118 | 6.5 | -912 | -5.7 | -1810 | -6.8 | -562 | -19.2 |
| 2050 | SSP126 | 8693 | 2.9 | 2621 | 20.0 | 2512 | 7.7 | 152 | 1.0 | 619 | 2.3 | -25 | -0.9 |
|  | SSP245 | 6799 | 2.3 | 804 | 6.1 | 2277 | 7.0 | 54 | 0.3 | -199 | -0.8 | -54 | -1.9 |
|  | SSP370 | 1056 | 0.4 | 796 | 6.1 | 2465 | 7.6 | -655 | -4.1 | -3334 | -12.6 | -988 | -33.7 |
|  | SSP585 | -3853 | -1.3 | -1242 | -9.5 | 2371 | 7.3 | -1604 | -10.1 | -3045 | -11.5 | -521 | -17.8 |
| 2070s | SSP126 | 6964 | 2.3 | 1191 | 9.1 | 2491 | 7.6 | 170 | 1.1 | -43 | -0.2 | -68 | -2.3 |
|  | SSP245 | 8923 | 3.0 | -199 | -1.5 | 2614 | 8.0 | -174 | -1.1 | -811 | -3.1 | -224 | -7.7 |
|  | SSP370 | -426 | -0.1 | -2208 | -16.8 | 2407 | 7.4 | -1343 | -8.4 | -5796 | -21.9 | -1046 | -35.7 |
|  | SSP585 | 4488 | 1.5 | -3099 | -23.6 | 2922 | 8.9 | -1879 | -11.8 | -5767 | -21.8 | -768 | -26.2 |
| 2090s | SSP126 | 10600 | 3.5 | 1749 | 13.3 | 2447 | 7.5 | 14 | 0.1 | -80 | -0.3 | 29 | 1.0 |
|  | SSP245 | 13411 | 4.5 | 749 | 5.7 | 2712 | 8.3 | -478 | -3.0 | -1405 | -5.3 | -138 | -4.7 |
|  | SSP370 | 10631 | 3.6 | -2625 | -20.0 | 2686 | 8.2 | -1636 | -10.3 | -6136 | -23.2 | -1504 | -51.3 |
|  | SSP585 | 11187 | 3.7 | -6147 | -46.9 | 3110 | 9.5 | -2719 | -17.1 | -7009 | -26.5 | -1446 | -49.3 |

Source: Model simulations

**References**

1 MFA. Coffee production in Ethiopia. *The 4th World Coffee Conference in Addis Ababa, Ministry of Foreign Affairs of Ethiopia, Addis Ababa, Ethiopia* (2016).

2 Mengistie, G. in *Extending the Protection of Geographical Indications: Case studies of Agricultural Products of Africa* Vol. 15 (eds M Blakeney, T Coulet, Getachew Mengistie, & M.T Mahop) 150 (Routledge, 2011).
